# Salivary Gland Manifestations of Long COVID: A Case Report and Literature Review

**DOI:** 10.3390/healthcare14162558

**Published:** 2026-08-15

**Authors:** Roman Skrypnyk, Maksym Skrypnyk, Margarita Skikevych, Igor Skrypnyk, Tetiana Yatsenko, Tatiana Petrushanko, Axel Spahr, Chun Xu, Satoshi Takahashi, Taro Osada, Shlomo Berkovsky, Koichi Hattori, Beate Heissig

**Affiliations:** 1 Department of Internal Medicine №1, Poltava State Medical University, 23 Shevchenko Street, 36011 Poltava, Ukraine; fatrich49@gmail.com (R.S.); inskrypnyk@gmail.com (I.S.); 2 Centre for Health Informatics, Australian Institute of Health Innovation, Faculty of Medicine, Health and Human Sciences, Macquarie University, 75 Talavera Road, Sydney, NSW 2109, Australia; shlomo.berkovsky@mq.edu.au; 3 Charles Perkins Centre, The University of Sydney, Johns Hopkins Drive, Sydney, NSW 2050, Australia; chun.xu@sydney.edu.au; 4 Sydney Dental School, Faculty of Medicine and Health, The University of Sydney, 2 Chalmers St., Sydney, NSW 2010, Australia; axel.spahr@sydney.edu.au; 5 Department of Surgical Dentistry and Maxillofacial Surgery, Poltava State Medical University, 23 Shevchenko Street, 36011 Poltava, Ukraine; skikevich1959@gmail.com; 6 Juntendo Biomedical Research Core Facilities, Juntendo University, Graduate School of Medicine, 2-1-1 Hongo, Bunkyo-Ku, Tokyo 113-8421, Japan; tetyanaa.yatsenko@gmail.com; 7 Department of Enzymes Chemistry and Biochemistry, Palladin Institute of Biochemistry of NAS of Ukraine, 9 Leontovich St., 01054 Kyiv, Ukraine; 8 Department of Therapeutic Dentistry, Poltava State Medical University, 36011 Poltava, Ukraine; petrusankotatana@gmail.com; 9 Division of Clinical Precision Research Platform, The Institute of Medical Science, The University of Tokyo, 4 Chome-6-1, Minato-Ku, Tokyo 108-8639, Japan; radius@ims.u-tokyo.ac.jp; 10 Department of Gastroenterology, Juntendo University Urayasu Hospital, 2-1-1 Tomioka, Urayasu-shi, Chiba 279-0021, Japan; otaro@juntendo.ac.jp; 11 Center for Genome and Regenerative Medicine, Graduate School of Medicine, Juntendo University, 2-1-1 Hongo, Bunkyo-Ku, Tokyo 113-8421, Japan; khattori@juntendo.ac.jp; 12 Department of Hematology/Oncology, The Institute of Medical Science, The University of Tokyo, 4-6-1, Minato-Ku, Tokyo 108-8639, Japan

**Keywords:** salivary glands, aged, COVID-19, ductal spasm, xerostomia, frailty, sialodochitis, long COVID-19 syndrome

## Abstract

**Highlights:**

**What are the main findings?**

- This case report describes a delayed ductal-only phenotype of long COVID, predominantly affecting the submandibular glands, that developed months after repeated SARS-CoV-2 infections.
- Marked salivary duct dilatation, xerostomia, facial swelling, and pain resolved completely after two months of conservative management, without surgery.

**What are the implications of the main findings?**

- Clinicians should consider post-COVID-19 sialodochitis in the differential diagnosis of salivary gland swelling and xerostomia, especially in older adults with a history of SARS-CoV-2 infection.
- Early recognition and conservative treatment may prevent progression to bacterial or necrotizing sialadenitis, thereby reducing the need for systemic antibiotics or invasive procedures in frail elderly patients.

**Abstract:**

**Background:** While acute coronavirus disease 2019 (COVID-19) infections of the oral cavity, with clinical signs such as taste loss, dry mouth, and mucosal lesions, are well-established health problems, involvement of the oral cavity as a sign of long COVID is poorly understood. One of the organs in the oral cavity that is prone to the virus is the salivary gland. **Case presentation**: We describe the case of an elderly patient with severe generalized xerostomia and left-sided facial swelling involving the submandibular gland, an uncommon presentation, as the parotid gland is typically involved COVID-19 infections. Ultrasound demonstrated marked ductal dilation without glandular tissue changes. The absence of other causes of sialodochitis, together with a history of repeated COVID-19 infections, led to the diagnosis of post-COVID sialodochitis. Management options were limited by the patient’s frailty and polypharmacy. Nevertheless, conservative symptomatic treatment led to clinical recovery, with no evidence of progression to bacterial or necrotizing sialadenitis. **Conclusions:** This case demonstrates that COVID-19 infection and its history should be included in the differential diagnosis of sialadenitis. Along with the clinical case presentation, a narrative review of similar studies on COVID-19-associated salivary gland disease was conducted, spanning molecular and post-mortem evidence, acute clinical cases, and post-COVID sequelae.

## 1. Introduction

The term long COVID (post-COVID-19 condition) refers to symptoms occurring in individuals with a history of probable or confirmed Severe Acute Respiratory Syndrome Coronavirus 2 (SARS-CoV-2) infection, typically appearing three months after disease onset, lasting at least two months, and not explained by an alternative diagnosis. Long COVID encompasses a wide range of symptoms and conditions that may improve, worsen, or persist. Long COVID is likely underdiagnosed and misdiagnosed, and salivary gland (SG) involvement remains underreported.

Reports of SG disease as a late manifestation of COVID-19 are currently scarce [1]. In the case presented below, we aim to contribute to the literature by describing a delayed ductal-only phenotype of long COVID, predominantly affecting the submandibular glands, that developed months after repeated SARS-CoV-2 infections, in an elderly palliative patient.

In frail older adults with multiple comorbidities, management is often limited by polypharmacy, age-related factors, and restricted treatment options. Reduced salivary flow from sialodochitis increases the risk of necrotizing sialadenitis due to salivary stasis and retrograde bacterial invasion from the oral cavity. When necrotizing sialadenitis develops in older adults, surgical management carries a high mortality risk, underscoring the importance of early diagnosis and noninvasive treatment. In our case, we demonstrated that early recognition and conservative treatment may prevent progression to bacterial or necrotizing sialadenitis, thereby reducing the need for invasive procedures in frail elderly patients.

## 2. Case Presentation

**Figure 1:**
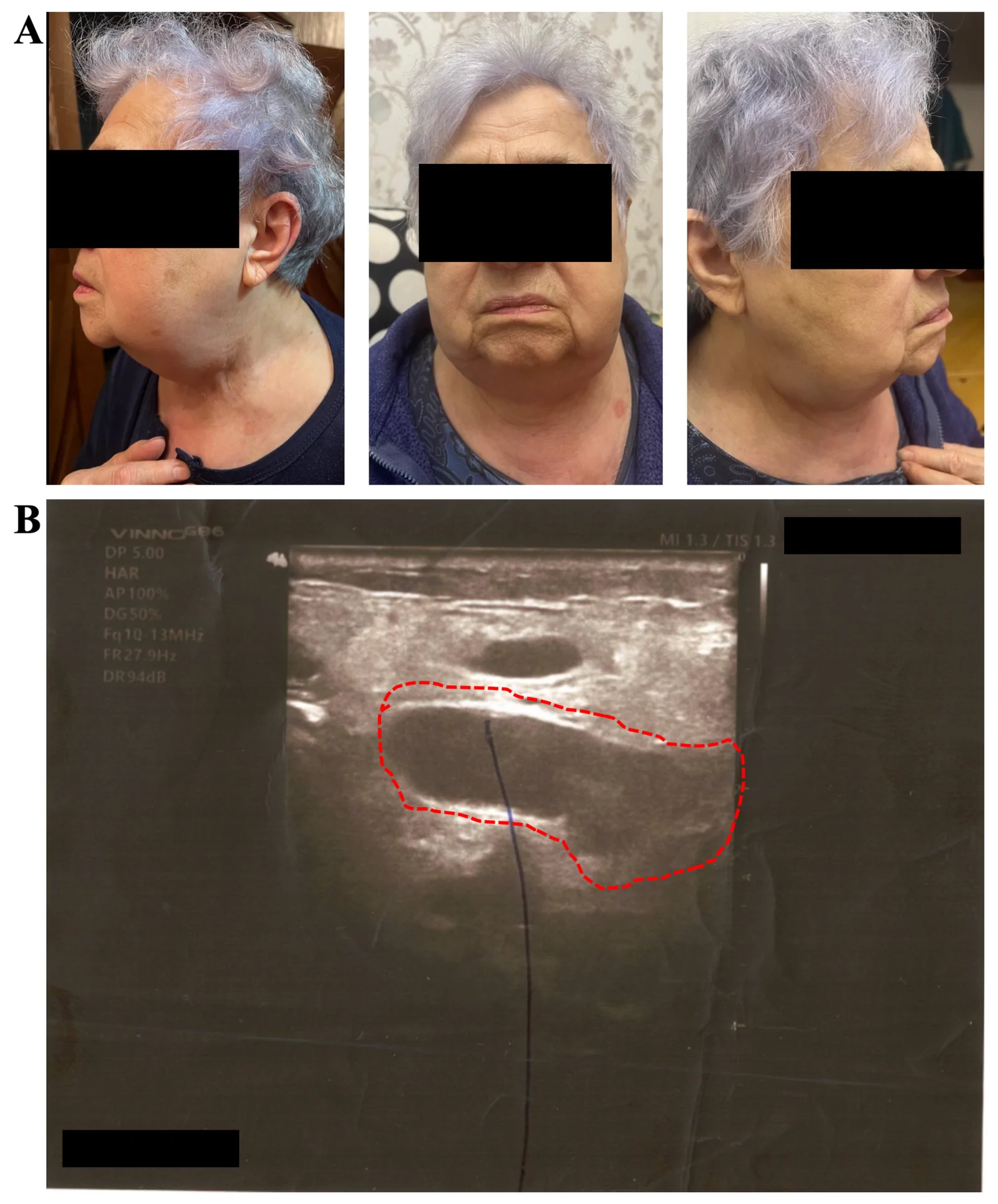
(**A**) Clinical presentation of the patient on the morning of the first day of symptom onset, showing facial asymmetry due to swelling on the left side of the face. (**B**) Ultrasound image of the left submandibular salivary gland on day 6 after symptom onset, demonstrating marked ductal dilatation up to 0.8 cm (approximately 13.33 times larger than the right submandibular duct), highlighted by the red dotted line.

An 80-year-old woman presented to our maxillofacial surgery department with left-sided facial swelling involving the cheek and submandibular region (Figure 1A), along with excruciating, bursting pain in all major SG. The symptoms were accompanied by xerostomia, difficulty eating, and generalized fatigue.

Initial laboratory tests showed a normal white blood cell count (8.45 × 10^9^/L) and lymphopenia (9.1%; 0.77 × 10^9^/L), neutrophilia (83.2%; 7.03 × 10^9^/L), monocytes (6.7%; 0.67 × 10^9^/L), and eosinophils (1%; 0.08 × 10^9^/L), as determined by an automated hematology analyzer. Increased band neutrophils (7%) and segmented neutrophils (73%), as determined by manual count.

Inflammatory markers were elevated, with C-reactive protein at 48 mg/L and an erythrocyte sedimentation rate of 46 mm/h. Venous blood gas analysis demonstrated marked alkalemia (pH 7.61). As no additional blood gas parameters (e.g., pCO_2_, bicarbonate, or base excess) were measured, the underlying etiology could not be further investigated. Possible explanations include respiratory alkalosis secondary to hyperventilation caused by severe pain or metabolic alkalosis associated with dehydration due to reduced oral intake resulting from difficulty eating and drinking. However, other causes cannot be excluded. All other biochemical parameters, including coagulation profiles, were within reference limits.

Medication history revealed long-term treatment with the antihypertensive drug moxonidine (an alpha-2/imidazoline receptor agonist) at 600–800 µg/day for the past 10 years. The patient is resistant to sartans and angiotensin-converting enzyme inhibitors and has a history of severe, poorly controlled hypertension. She exceeds the prescribed 600 µg daily dose of moxonidine only during hypertensive crises.

The patient’s past medical history included recurrent COVID-19 infections at 48, 12, and 6 months before presentation, with lung involvement in the 48-month episode, despite receiving three doses of the COVID-19 vaccine. SG involvement was not observed during the prior COVID-19 episodes. A detailed timeline of the patient’s clinical course is shown in Table 1.

Based on the inflammatory parameters, the following differential diagnoses were considered: epidemic parotitis, sialolithiasis, COVID-19-associated sialadenitis, sialodochitis fibrinosa (Kussmaul disease), drug-induced (moxonidine), and acute bacterial sialadenitis. Epidemic parotitis was ruled out by a negative PCR for Orthorubulavirus parotitis; sialolithiasis was excluded on CT, which showed no salivary stones. Kussmaul disease was excluded due to the absence of a typical allergic component. Although moxonidine can cause xerostomia, given the long-term use, its role in the current clinical situation was considered less likely. Acute bacterial sialadenitis was ruled out based on the clinical presentation. Likewise, a negative SARS-CoV-2 PCR test ruled out an active COVID-19 infection.

Ultrasonography of the SG revealed sialectasis: duct diameter of 0.8 cm in the left submandibular gland (normal up to 0.3 cm) (Figure 1B), with the gland measuring 4.5 × 3.6 cm; the right gland measured 3.2 × 1.7 cm with a duct of 0.06 cm. The right parotid was 3.3 × 1.2 cm and indurated, with duct diameter up to 0.07 cm; the left parotid was 4.7 × 3.0 cm with a duct up to 0.14 cm. No ductal obstructions from stones, tumors, or inflammation. Enlarged lymph nodes were present along the left neck (up to 1.5 cm) and smaller on the right (up to 0.6 cm).

The presumptive diagnosis was post-COVID-19 generalized sialodochitis, with COVID-19 considered a potential triggering factor against a background of multiple risk factors for xerostomia, including advanced age and moxonidine-induced xerostomia, especially as the patient’s moxonidine daily intake exceeded the licensed maximum of 600 µg/day. Treatment included increased fluid intake and SG massage. Avoidance of spicy, sour, and salty foods was recommended because the patient presented with xerostomia, and these foods could irritate the oral mucosa. This recommendation was made before spasmolytic therapy became effective.

Salivary stimulation was expected to worsen severe pain, as the excretory duct was in spasm and could not be probed. Increased saliva production without adequate drainage due to ductal obstruction would likely have exacerbated the patient’s symptoms. The patient received 80 mg of drotaverine hydrochloride and 200 mL of Reosorbilact IV, which consists of: sorbitol 60.0 mg, sodium lactate solution 19.0 mg, sodium chloride 6.0 mg, calcium chloride dihydrate 0.1 mg, potassium chloride 0.3 mg, and magnesium chloride hexahydrate 0.2 mg, compared with Ringer’s solution, which contains only electrolytes. Due to the presence of sorbitol, Reosorbilact IV improves capillary flow and has an anti-aggregating effect. Topically, Vishnevsky liniment was applied, which contains birch tar, xeroformium (bismuth tribromophenolate), and castor oil, and is used as a topical medication for wounds, burns, and suppuration, as it causes local irritation, has mild antiseptic properties, and enhances blood flow. SG duct probing failed due to papillary spasm. Symptoms resolved after two months of treatment with spasmolytics, active SG massage (often retrieving scant saliva), local applications of Vishnevsky liniment, and increased water intake. This case highlights the favorable outcome of conservative management for chronic sialodochitis, possibly a long-term complication of COVID-19, and for preventing bacterial sialadenitis associated with reduced salivary flow.

The patient expressed relief that conservative management was chosen over surgery and was satisfied with the gradual recovery, which led to symptom resolution over approximately two months without surgical intervention or antibiotics. A limitation of this report is the inability to distinguish the effects of the administered therapies from the natural course of the disease. Spontaneous resolution, hydration, and SG massage may have played a significant role in the patient’s recovery. Furthermore, several treatments used in this case, including drotaverine, Reosorbilact, and Vishnevsky liniment, lack an established evidence base for sialodochitis.

### Patient Perspective

“The swelling and the bursting pain in my face and under my jaw frightened me, and the constant dry mouth made it hard to eat and speak. Because of my age and the other medicines I take, I was relieved that the doctors chose a gentle treatment instead of an operation. The recovery was slow; it took about two months, but the pain settled and the swelling went down, and I was glad to avoid surgery and antibiotics.”

## 3. Discussion

Currently, information on how SARS-CoV-2 affects the SGs, including clinical presentation and management after acute infection, is limited. This poses a significant challenge for clinicians, particularly when treating frail patients. SGs are recognized as target organs for the initial manifestation of COVID-19, especially the ductal epithelium, luminal surfaces, and serous acini, due to high expression of angiotensin-converting enzyme 2 and TMPRSS receptors [2,3]. SARS-CoV-2-infected oral mucosal epithelial cells are shed into saliva [2]. Therefore, at the beginning of the pandemic, medications that induce hyposalivation were considered potentially protective because they could reduce viral transmission through saliva [4]. Some clinical evidence suggests that long COVID-19 complications may include SG parenchymal atrophy, ductal rupture, and fibrosis. Viral persistence and cellular degeneration have been observed in these tissues, as shown in autopsy studies of COVID-19 patients who died from unrelated causes [5].

Sialadenitis in patients with SARS-CoV-2 is an uncommon manifestation of COVID-19 [5]. A recent systematic review found that males and females were equally affected, with a mean age of 35.6 years. The parotid SG was most commonly involved, and 45% of cases were bilateral. Involvement of the submandibular and sublingual SG was reported in only one patient [6]. Parotitis was associated with COVID-19 detection, and it was identified in 22% of cases after COVID-19 had been diagnosed [6]. Management is limited to symptomatic treatment, with antibiotics being used in 12% of cases to prevent bacterial superinfection [6]. Another large retrospective study found that changes affecting the SGs occurred in 11% of people, including 6.8% of women and 4.2% of men based on the analysis of medical records of the study population of 1256 patients. Xerostomia was found in 60 people (47 women and 13 men) [7].

This case is unusual because it highlights typical features of sialodochitis, characterized by changes in the SG ducts without alterations in the glandular tissue itself. Sialodochitis is often associated with salivary duct strictures and salivary stones (sialolithiasis). A specific form of sialodochitis, eosinophilic sialodochitis (Kussmaul disease), is linked to allergies, which was not observed in our patient [8]. Salivary duct probing was not possible in this case due to severe spasm of the excretory duct papilla, even though it is usually the treatment of choice for sialodochitis and provides significant symptom relief. Instead, we implemented a gentle conservative medical treatment approach, including spasmolytics, infusion therapy, and local anti-inflammatory applications. Additionally, the patient’s medications, specifically the use of an alpha-2/imidazoline receptor agonist, may have influenced the clinical presentation of the disease, particularly in the context of concurrent COVID-19 infection. Therefore, these medications were paused during treatment, and the first signs of improved salivary flow were observed on the eighth day after discontinuation.

### Literature Review: COVID-19-Associated SG Disease

To contextualize this case within the published literature, we conducted a narrative review. PubMed/MEDLINE was searched using free-text combinations of (“COVID-19” OR “SARS-CoV-2”) with “salivary gland”, “saliva”, “sialadenitis”, “parotitis”, “xerostomia”, “sialodochitis”, and “ACE2”. Studies were selected purposively to span molecular, post-mortem, clinical, and experimental evidence; no systematic review methodology was used (Table 2). Bioinformatic and single-cell studies established that ACE2 and TMPRSS proteases are expressed in oral and SG ductal and acinar epithelium, providing the molecular basis for viral tropism [2,3]. Post-mortem analysis confirmed the presence of SARS-CoV-2 RNA and replicating virus in SG ducts and acini, with detection in 60% of SG samples from fatal cases [5]. Clinically, reported SG manifestations have predominantly involved the parotid gland during active infection, presenting either as true sialadenitis or, in one series, as intraparotid lymphadenitis that produced a parotitis-like picture [9,10]. Submandibular involvement is uncommon, and sublingual involvement was first described only in 2022 [11]. Post-acute sequelae include persistent SG ectasia in a large survivor cohort followed for a median of 104 days [12] and histatin-5 depletion with Th17/IL-23 activation resembling Sjogren’s syndrome [13]. Experimental data show that the spike protein alone can induce Sjogren-like SG dysfunction independently of ACE2 [14]. Xerostomia and other oral symptoms are also common in mild-to-moderate disease [15]. Against this background, the presented case is distinctive: a delayed, submandibular-predominant ductal (sialodochitis) phenotype that develops months after repeated infections and is managed conservatively without antibiotics (Table 2).

## 4. Conclusions

This case illustrates a prolonged recovery after sialodochitis, potentially triggered by recurrent COVID-19 infections and long-term post-COVID effects. Unlike most previously reported long COVID-19 cases, in which the parotid gland is typically involved, our patient presented with submandibular gland involvement. We report that a symptomatic approach was well accepted by the frail elderly patient in this case. Although suppurative sialadenitis—bacterial infections of the SG—is common in older adults and associated with high mortality, no such complications developed, and the patient recovered with symptomatic treatment. Importantly, antibiotic therapy was not necessary. Further studies, including those on long-term viral storage and potential shedding, and ductus damage due to COVID-19 infection will be necessary to improve treatment strategies.

## Figures and Tables

**Table 1:** Clinical course from recurrent COVID-19 infections to complete resolution of generalized sialodochitis after two months of conservative therapy.

Time Point	Clinical Events and Findings	Interventions/Decisions
48 months before presentation	First COVID-19 infection with lung involvement (1 of 3 PCR-confirmed episodes); patient had received 3 vaccine doses.	No SG swelling observed.
12 and 6 months before presentation	Second and third COVID-19 infections; no SG swelling during either episode.	Routine recovery; no salivary-gland-directed treatment.
Day 1 (symptom onset)	Acute left-sided facial and submandibular swelling (Figure 1A) with bursting pain in all major SGs; xerostomia, difficulty eating, fatigue.	Presentation to maxillofacial surgery; increased fluids given to the patient, avoidance of spicy/sour/salty foods, gland massage.
Days 1–6 (diagnostic of the condition)	WBC 8.45 × 10^9^/L, lymphopenia 9.1%; 0.77 × 10^9^/L, neutrophilia (83.2%; 7.03 × 10^9^/L), monocytes (6.7%; 0.67 × 10^9^/L), and eosinophils (1%; 0.08 × 10^9^/L) [automated cell count]; 7% band neutrophils and 73% segmented neutrophils [manual count]; CRP 48 mg/L, ESR 46 mm/h; venous blood gas analysis pH 7.61; Orthorubulavirus and SARS-CoV-2 PCR negative; CT: no sialolith.	Differential diagnosis: mumps, sialolithiasis, Kussmaul disease, bacterial or drug-induced (moxonidine) sialadenitis and acute COVID-19-associated sialadenitis excluded.
Day 6	Ultrasound: left submandibular duct 0.8 cm (≈13.33× the contralateral duct), gland 4.5 × 3.6 cm, no obstruction; enlarged left cervical nodes up to 1.5 cm.	Diagnosis: post-COVID-19 generalized sialodochitis. Drotaverine 80 mg, Reosorbilact 200 mL IV, topical Vishnevsky liniment. Duct probing attempted but failed (papillary spasm).
Weeks 1–8	Gradual reduction in pain and swelling; only scant saliva expressed on massage, initial improvement of salivation.	Continued spasmolytics, active gland massage, Vishnevsky liniment, increased water intake.
≈2 months	Full resolution of symptoms; no progression to suppurative or bacterial sialadenitis.	Conservative therapy concluded; no antibiotics required.

**Table 2:** Summary of key studies on COVID-19-associated SG disease, from molecular and post-mortem evidence to acute clinical cases and post-COVID sequelae.

Study (Author, Reference)	Year	Study Type	Gland(s)	COVID-19 Timing	Key Finding	Management	Outcome
Xu H et al. [3].	2020	Bioinformatic/RNA-seq	Oral mucosa (esp. tongue); SG not specifically resolved	Baseline (pre-clinical)	ACE2 expressed on oral epithelium, highly enriched in tongue; rationale for oral SARS-CoV-2 susceptibility	N/A	Oral ACE2 map established
Huang N et al. [2].	2021	scRNA-seq atlas + autopsy/outpatient validation	Minor & major SGs, oral mucosa	Active + autopsy	ACE2/TMPRSS in SG duct & acinar epithelia; replicating virus in ducts/acini; chronic focal lymphocytic sialadenitis, atrophy, ductal rupture, fibrosis	N/A	SG ducts/acini confirmed as infection and injury site
Matuck BF et al. [5].	2021	Post-mortem cohort (24 fatal cases)	Parotid, submandibular, minor SGs	Fatal acute COVID-19 (autopsy)	SARS-CoV-2 RNA in 60% of SG samples (75% of cases); viral particles in ductal/acinar cells; intense ACE2/TMPRSS in duct and serous acini	N/A (autopsy)	Viral persistence/replication in SG confirmed
Chern A et al. [9].	2020	Case series (2 patients)	Case 1 parotid; Case 2 bilateral parotid + submandibular	Concurrent active COVID-19	First documented COVID-19 parotid/submandibular sialadenitis; CT gland enlargement; no abscess or sialolith	IV then oral antibiotics, sialagogues, hydration	Resolution
Lechien JR et al. [10].	2020	Case series (3 women)	Parotid (unilateral/bilateral)	Onset or course of active COVID-19	MRI showed intraparotid lymphadenitis producing a parotitis-like picture, not primary sialadenitis	Paracetamol (10–14 days)	Parotitis resolved; persistent anosmia
Gherlone EF et al. [12].	2021	Retrospective/prospective cohort (122 survivors)	Major SGs	Post-COVID (median 104 days after discharge)	SG ectasia in ≈38–43%; oral abnormalities in 83.6%; ectasia linked to CRP/LDH and antibiotic use	Conservative	Residual oral/SG damage persisted in most survivors
Maegawa K, Nishioka H. [11].	2022	Case report	Bilateral parotid + sublingual	Acute severe COVID-19 (ICU; swelling day 6)	First reported sublingual SG and parotid bilateral involvement; and fat stranding on CT; clear saliva, no pus, no duct dilation	Supportive (dexamethasone, tocilizumab, ceftriaxone)	Died of respiratory failure (day 14)
Alfaifi A et al. [13].	2022	Case report + salivary analysis	Major SGs (functional)	Post-COVID (months after recovery)	≈92% reduction in salivary histatin-5; Th17/IL-23 activation; oral dysesthesia/dysgeusia; salivary flow normal	Topical lidocaine/diphenhydramine rinse	≈25% symptom improvement; dysgeusia resolved
Felix FA et al. [14].	2025	Experimental (C57BL/6 mice, intra-SMG injection)	Submandibular gland (model)	Mechanistic	Spike (not envelope) protein alone reduced salivary secretion, induced leukocyte foci in ≈two-third of mice (ANA in ≈one-third); Sjogren-like, ACE2-independent	N/A	Spike sufficient for Sjogren-like SG pathology
Alkharobi HE et al. [15].	2024	Cross-sectional (mild–moderate COVID-19)	Oral cavity/SG function	Acute mild–moderate COVID-19	Common oral symptoms: lip dryness, gingivitis, tongue changes, taste loss; no difference by tobacco use	Symptomatic	Oral symptoms frequent; routine oral screening advised
Present case	2025	Case report	All major SGs; left submandibular most severe	Post-COVID (6 months after last of 3 infections)	Generalized sialodochitis; ductal dilation 0.8 cm; excretory papillary spasm; no parenchymal change	Conservative (spasmolytics, infusion, gland massage, Vishnevsky liniment; no antibiotics)	Full resolution at 2 months; no bacterial sialadenitis

## Data Availability

All original data related to this study was presented in the manuscript. More information, such as original results of medical investigations which contain personal information on the patient, by which she can be identified, was not presented and is available from the corresponding authors on reasonable request.

## Abbreviations

The following abbreviations are used in this manuscript:

COVID-19	coronavirus disease 2019
SARS-CoV-2	severe acute respiratory syndrome coronavirus 2
SG	salivary gland
IV	intravenous
